# Comparison of efficacy and complications of endoscopic and percutaneous biliary drainage in malignant obstructive jaundice: a systematic review and meta-analysis

**DOI:** 10.1186/s40644-017-0129-1

**Published:** 2017-10-16

**Authors:** Feng Duan, Li Cui, Yanhua Bai, Xiaohui Li, Jieyu Yan, Xuan Liu

**Affiliations:** Department of Interventional Radiology, the General Hospital of Chinese People’s Liberation Army, Beijing, 100853 China

**Keywords:** Endoscopic biliary drainage, Percutaneous biliary drainage, Malignant obstructive jaundice

## Abstract

**Background:**

Malignant obstructive jaundice is a common problem in the clinic. Currently, the generally applied treatment methods are percutaneous transhepatic biliary drainage (PTBD) and endoscopic biliary drainage (EBD). Nevertheless, there has not been a uniform conclusion published on either efficacy of the two types of drainage or the incidence rate of complications. Therefore, we conducted a systematic review and meta-analysis of studies comparing endoscopic versus percutaneous biliary drainage in malignant obstructive jaundice, to determine whether there is any difference between percutaneous and endoscopic biliary drainage, with respect to efficacy and incidence rate of overall complications.

**Methods:**

The enrolled studies contain a total of three randomized controlled trials and eleven retrospective studies, which together encompass 2246 patients with PTBD and 8100 patients with EBD.

**Results:**

Our analysis indicates that there is no difference between PTBD and EBD with regard to therapeutic success rate (%), overall complication (%), intraperitoneal bile leak, 30-day mortality, sepsis, or duodenal perforation (%). Cholangitis and pancreatitis after PTBD were lower than after EBD, with odds ratios (OR) of 0.48 (95% confidence interval (CI), 0.31 to 0.74) and 0.16 (95% CI, 0.05 to 0.52), respectively. Incidences of bleeding and tube dislocation for PTBD were higher than EBD, OR of 1.81 (95% CI, 1.35 to 2.44) and 3.41 (95% CI, 1.10 to 10.60).

**Conclusions:**

This meta-analysis indicates certain advantages for both PTBD and EBD. In the clinical practice, it is advised to choose specifically either PTBD or EBD, based on location of obstruction, purpose of drainage (as a preoperative procedure or a palliative treatment) and level of experience in biliary drainage at individual treatment centers.

## Background

Malignant obstructive jaundice can occur following pancreatic cancer, Ampulla of Vater, or hilar cholangiocarcinoma, etc. [1]. If it is not handled in a timely manner, it may cause lots of adverse events such as cholangitis, delay tumor treatment, reduce quality of life and increase death rate, etc. Successful biliary drainage can significantly improve prognosis in patients with malignant obstructive jaundice [2]. To date, the generally applied clinical biliary drainage methods are percutaneous transhepatic biliary drainage (PTBD) and endoscopic biliary drainage (EBD). Regarding efficacy of the two types of drainage and incidence rate of complications, interventional radiologists and gastroenterologists hold different opinions, resulting in diverging opinions on treatment approach [3, 4]. Therefore, we conducted a meta-analysis, to determine whether there is a difference with respect to efficacy and incidence rate of overall complications between PTBD and EBD.

## Methods

### Search strategy

A comprehensive search of literature was conducted on Pubmed, EMBASE database and Cochrane Central Register of Controlled Trials to identify articles published until February 28th of 2017, on comparing PTBD and EBD in the management of malignant biliary tract obstruction. The search index terms were (a) ‘pancreatic neoplasms’ (medical subject heading, MeSH) OR ‘cholangiocarcinoma’, (MeSH) OR malignant biliary obstruction (title/abstract, TIAB), and with (b) ‘drainage’ (MeSH) OR ‘percutaneous transhepatic biliary drainage’ (TIAB) OR PTBD (TIAB) OR ‘endoscopic retrograde biliary drainage’ (TIAB) OR ‘ERCP (endoscopic retrograde cholangiopancreatography)’ (TIAB), and with (c) ‘complications’ (Subheading) OR ‘adverse event’ (Subheading) OR ‘mortality’ (MeSH) OR ‘therapeutics’ (MeSH). We identified additional publications by cross-checking references of the retrieved full-text articles.

### Study selection

Study selection criteria were (a) written in English, (b) carried out in patients with malignant biliary obstruction, and (c) comparing PTBD and EBD for palliation of biliary obstruction. Studies were excluded when, (a) evaluations were based on only one arm of PTBD or EBD, (b) focused on PTBD after ERCP failure instead of primary PTBD, or (c) lacking data on complications, therapeutic success or drainage-related mortality. For articles reporting duplicate data, the one with the most detailed data set was selected.

Two readers independently reviewed all retrieved titles and abstracts to identify potentially eligible studies according to the selection criteria listed above, and any divergence was resolved after discussion and consensus was reached.

### Data extraction

Two readers independently extracted data from the enrolled studies into a unified data extraction form, including information on author name, study year, study area, sample size, cancer type, drainage method, overall complication rate and incidence of each complication, therapeutic success rate, drainage-related mortality rate, drainage patency, length of stay and survival outcomes. Data for endoscopic drainage methods (endoscopic nasobiliary drainage and endoscopic biliary sphincterotomy) were combined prior to comparison with data for PTBD. Patients requiring conversion from one form of drainage to another were subsequently analyzed using intention-to-treat analysis. All available and relevant qualitative study measures were combined by tabulation of each drainage group. Upon any contradictory data extraction, the two readers discussed the data to reach a consensus.

### Quality assessment

The quality of enrolled cohort studies was assessed by using the 9-star Newcastle-Ottawa Quality Assessment Scale, including 8 items on patient selection, comparability and outcome. Studies with 5 or more stars were interpreted as high-quality studies. The quality of enrolled randomized controlled trials (RCTs) were assessed using the 7-point Modified Jadad Score, including 7 items on randomization, allocation concealment, blinding and withdrawals, and dropouts. Studies with 4 or more points were interpreted as high-quality studies. Two readers independently conducted quality assessments, and divergence was resolved after discussion to reach consensus.

### Statistical methods

Odds ratios (ORs) were calculated for each outcome and depicted as categorical variables for every comparison. The Mantel-Haenszel method for fixed effects models was applied to all comparisons exhibiting no statistical heterogeneity to determine corresponding overall effect sizes and their confidence intervals (CIs). If statistical heterogeneity was noted, the DerSimonian and Laird method for random effects was used. Forrest plots were drawn to show the point estimates in each study in relation to the pooled summary estimate. The estimated width of the point in the Forrest plots indicates the assigned weight to that study. A two-sided *P* < 0.05 was considered to indicate statistical significance for pooled OR. The heterogeneity among studies was analyzed using a χ^2^-based test of homogeneity and the inconsistency index (I^2^) statistic was calculated. If the I^2^ statistic indicated that heterogeneity existed between studies (>10%), a random effects model was then used. Otherwise, fixed-effects models were used. A 0.10 significance level was used to identify heterogeneity across studies. Stratified analysis was further conducted according to study design (RCT or retrospective studies). Publication bias on the summary estimates was tested by both Begg adjusted rank-correlation test and the Egger regression asymmetry test. Funnel plots were also constructed to evaluate potential publication bias. A 0.05 significance level was used to identify publication bias. All statistical analyses were done using Review Manager 5.3 and STATA 10.0.

## Results

### Literature search and study selection

**Fig. 1:**
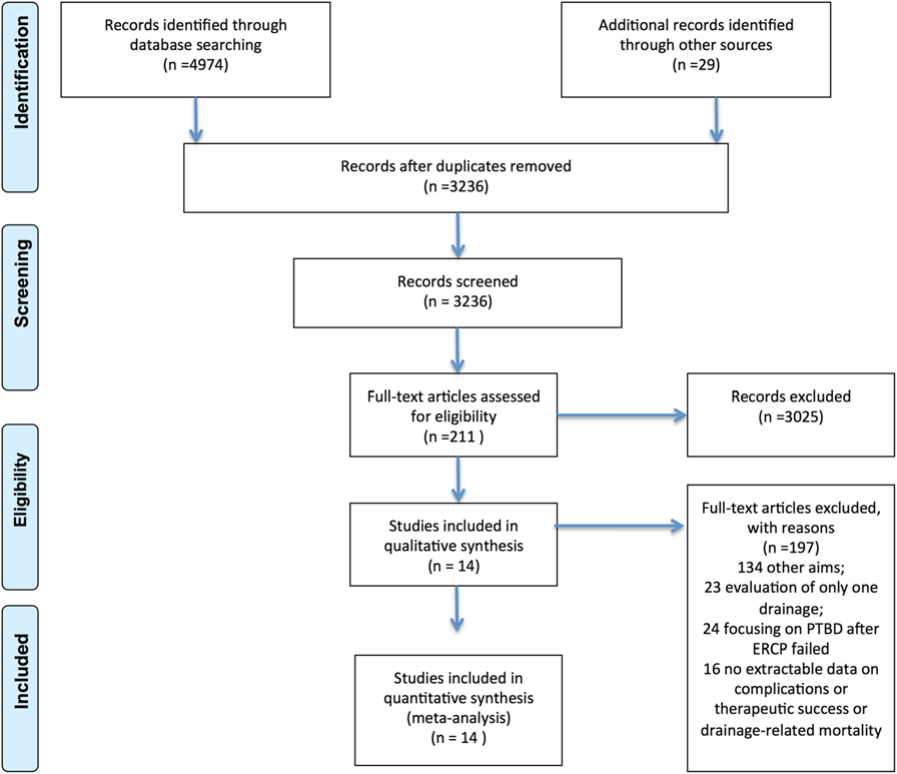
Flow Diagram. The enrolled studies represent a total of 3 RCTs and 11 retrospective studies, and encompass 2246 patients with PTBD and 8100 patients with EBD. After quality assessment, all studies were interpreted as high-quality studies. The characteristics of the studies are depicted in Table 1

The initial search identified 3236 titles and abstracts, of which 3025 publications were thereafter excluded, because they were not relevant with respect to drainage-related complications, therapeutic success or mortality. Therefore, a total of 211 publications were considered relevant and further evaluated. Finally, 14 publications were included in the meta-analysis (Fig. 1).

The enrolled studies represent a total of 3 RCTs and 11 retrospective studies, and encompass 2246 patients with PTBD and 8100 patients with EBD. After quality assessment, all studies were interpreted as high-quality studies. The characteristics of the studies are depicted in Table 1.

**Table 1:** Main characteristics of the included studies

Author	year	country	study design	No.Patients in study	No. PTBD	No. EBD	age(years, median or range)	Male (%)	Maligancy type	Quality assessment*
Speer AG	1987	Enghland	RCT	75	36	39	50–87	/	primary carcinoma of the pancreas, gallbladder, or bileducts	4
Piñol V	2002	Spain	RCT	54	28	26	73	74	primary carcinoma of the pancreas, gallbladder, or bileducts,or regional lymph node metastasis	5
Lee SH	2007	South Korea	retrospective	134(34 IPTBD)	66	34	67	69	Klatskin’s tumor	8
Saluja SS	2008	India	RCT	54	27	27	51	33	Gallbladder Cancer	5
Paik WH	2009	South Korea	retrospective	85	41	44	66	68	Hilar cholangiocarcinoma	8
Kloek JJ	2010	Netherlands	retrospective	101	11	90	61	69	Hilar cholangiocarcinoma	7
Choi J	2012	South Korea	retrospective	60	31	29	59	77	hepatocellular carcinoma	7
Walter T	2013	Canada	retrospective	129	42	87	66	60	Klatskintumors	8
Huang X	2015	China	retrospective	270(170 no PBD)	45	55(ENBD 18,ERBS 37)	58	71	extrahepatic bile duct cancer	7
Kim KM	2015	South Korea	retrospective	106	62	44	42–89	64	Perihilar cholangiocarcinomas	8
Inamdar S	2016	USA	retrospective	9135	1690	7445	70	50	pancreatic cancer or cholangiocarcinoma	6
Kishi Y	2016	Japan	retrospective	171	98	72	31–86	78	biliary tract cancer	7
Jo JH	2017	South Korea	retrospective	98	43	55(13 ENBD, 42 EBS)	63.5(29–82)	61	Klatskin tumor	8
Miura F	2017	Japan	retrospective	88	25	63	70(42–85)	70	extrahepatic bile duct cancer	7

*retrospective cohort studies was evaluated using 9-star Newcastle-Ottawa Quality Assessment Scale; RCT studies was evaluated using 7-point Modified Jadad Score

### Comparison between PTBD and EBD

Ten studies report therapeutic success rate, 13 studies report overall complications, and 9 studies report 30-day mortality. All studies provide incidence data of at least one kind of complication.

**Fig. 2:**
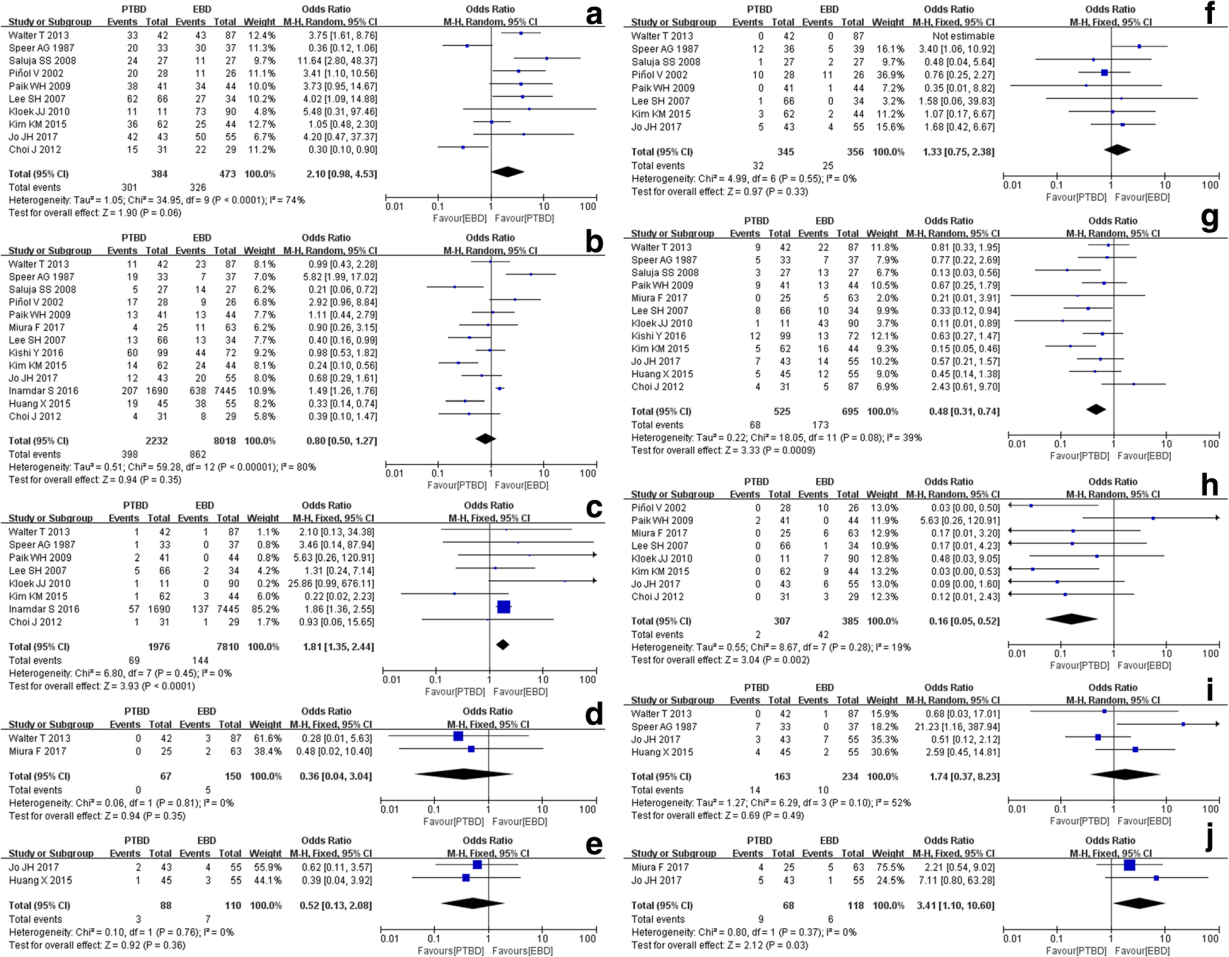
Forest plots (whole study). **a** therapeutic success rate; **b** overall complication; **c** bleeding; **d** duodenal perforation; **e** sepsis; **f** 30-day mortality rate; **g** cholangitis; **h** pancreatitis; **i** intra-peritoneal bile leak; **j** tube dislocation

PTBD was superior to EBD with respect to therapeutic success rate, incidence of overall complications, intraperitoneal bile leak, 30-day mortality, sepsis and duodenal perforation. PTBD demonstrated significant lower incidence of cholangitis and pancreatitis than EBD, with OR of 0.48 (95% CI, 0.31 to 0.74) and 0.16 (95% CI, 0.05 to 0.52) for cholangitis and pancreatitis, respectively. However, incidence of bleeding and tube dislocation for PTBD was significantly higher than EBD, with OR of 1.81 (95% CI, 1.35 to 2.44) and 3.41 (95% CI, 1.10 to 10.60) for bleeding and tube dislocation, respectively (Fig. 2).

**Fig. 3:**
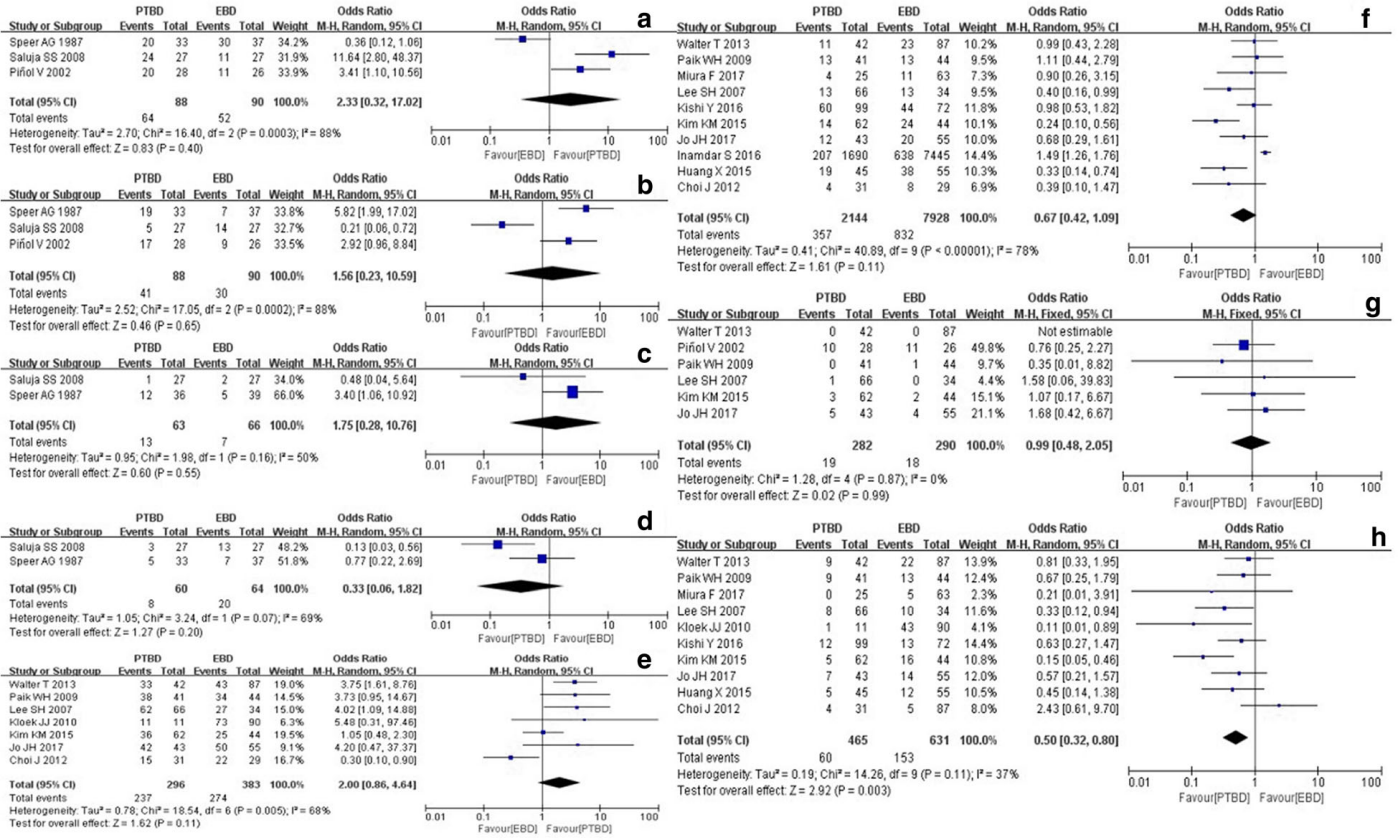
Forest plots (subgroup, divided by RCT and retrospective study) **a** rct-therapeutic success rate; **b** rct-overall complication; **c** rct-30-day mortality rate; **d** rct-cholangitis; **e** retrospective study-therapeutic success rate; **f** retrospective study-overall complication; **g** retrospective study-30-day mortality rate; **h** retrospective study - cholangitis

According to the stratified analysis of results from the RCT and retrospective studies (Fig. 3), there is no difference in cholangitis incidence rate between PTBD and EBD based on the RCT studies only, with an OR of 0.45 (95% CI, 0.16 to 1.21). However, based on the retrospective studies, PTBD group has a significantly lower incidence rate of cholangitis than EBD group, with an OR of 0.50 (95% CI, 0.32 to 0.80). Based on stratified analysis results of the RCT and retrospective studies, there is no difference between PTBD and EBD with respect to therapeutic success rate, overall complications or 30-day mortality rate.

### Publication bias

**Fig. 4:**
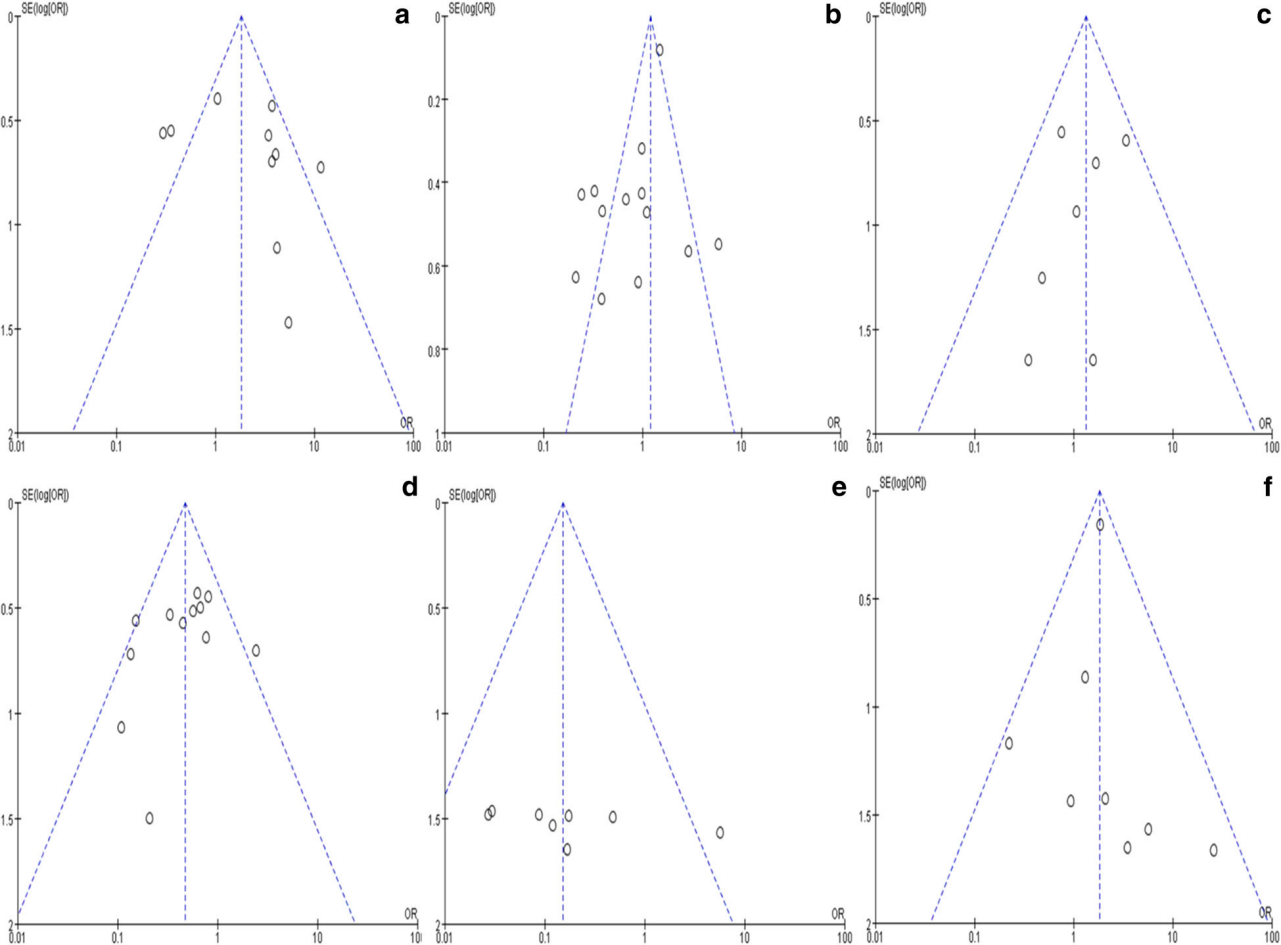
Funnel plots. **a** therapeutic success rate; **b** overall complication; **c** 30-day mortality rate; **d** Cholangitis; **e** pancreatitis; **f** bleeding

Analysis based on the Begg adjusted rank-correlation and the Egger regression asymmetry tests presents significant publication bias for therapeutic success rate and overall complications, which indicates that studies with smaller sample size are inclined to provide results favorable to PTBD. No publication bias was detected with respect to 30-day mortality or other complications (Fig. 4).

## Discussion

Although EBD has been performed much more frequently than PTBC in clinical practice on malignant obstructive jaundice, according to the results of our meta-analysis EBD did not show significant advantages over PTBD. PTBD resulted in better therapeutic success rate, and lower incidence of overall complications, intraperitoneal bile leak, 30-day mortality, sepsis and duodenal perforation, compared to EBD. With respect to cholangitis and pancreatitis, PTBD also showed to be a superior method.

Infection is one of the most common complication after biliary drainage. In combination with malignant biliary obstruction, patients are often in poor condition with complications such as low immunity and concurrent infection, which can have serious consequences [5–7]. Our study shows that incidence rate of cholangitis and pancreatitis was higher after EBD than after PTBD. There may be two reasons, which cause the high infection rate after EBD. Firstly, biliary drainage could be incomplete. Especially in patients with severe obstruction it is very difficult to ensure complete drainage of each biliary duct. Bacterial growth in the bile stasis after incomplete drainage causes secondary infection [8]. Secondly, incision of the duodenal papilla sometimes occurs during EBD, which damages the regular structure of duodenal papilla. As this structure prevents retrograde entering of biliary ducts or pancreatic duct by intestinal bacteria, which is the most important route of infection caused by biliary drainage. Damage to the duodenal papilla increases the chance of infection [9]. Application of antibiotics is another infection-related factor. Routine preventive administration of antibiotics is recommended prior to both PTBD and EBD [10]. Whether or not treatment with antibiotics should be continued after biliary drainage depends on completeness of drainage. And secondly, whether there is a concomitant infection pre-operation [11]. In stratified analysis, the disparity in results between the RCT and retrospective studies was observed for cholangitis incidence rate. Retrospective studies showed PTBD yields a significantly lower incidence rate of cholangitis than EBD, while RCTs showed similar rate of cholangitis between PTBD and EBD. RCT is usually recognized to provide higher level of evidence compared with retrospective study; however, there were only two RCTs (Speer 1987 and Saluja 2008) with small sample size (60 patients) providing data of incidence rate of cholangitis. On the contrary, there were ten more recent (published from 2007 to 2017) retrospective studies with a much larger sample size (465 patients) and assessed as high quality. Considering that there were improving and more efficacious percutaneous or endoscopic techniques in recent studies, we interpret the result from retrospective studies is more reliable and can reflect the real situation.

Taken together, our study shows that incidence of tube dislocation and bleeding after PTBD was higher compared to EBD [12, 13]. PTBD is also accompanied by a higher incidence rate of metastasis, which is an important complication. It has been reported that PTBD increases the incidence of metastasis after resection of distal cholangiocarcinoma and may shorten postoperative survival [14–16]. However, there are no comparison data available among the selected articles, which are suitable for meta-analysis.

Regarding the choice of biliary drainage methods, there are other factors which need to be considered, which however are not included in this study. First, regarding biliary drainage prior to surgery, PTBD (simple external drainage) is mainly performed, which is still under debate. There is a prospective randomized controlled trial currently ongoing [17], which hopefully provides convincing evidence on selection of biliary drainage methods prior to surgery. Second, quality of life after biliary drainage is also an important factor to consider. Theoretically, carrying an external drainage tube affects quality of life more than implantation of an internal stent. Nevertheless, based on a controlled study by Saluja et al., quality of life after PTBD was rated higher compared to the endoscopic biliary stent implantation group, according to World Health Organization Quality of Life physical and psychological scores at 1 and 3 months [18]. Although this study did not reach statistical significance, there was a trend towards a better quality of life after PTBD, which may be caused by the relatively high incidence rate of fever in the biliary stent implantation group. Regarding quality of life, large scale controlled studies are needed to conclude which drainage method is better. Third, accumulation of experience on PTBD is also important. It has been reported that, for biliary obstruction caused by cholangiocarcinoma, EBD is superior in medical centers that perform limited numbers of PTBD procedures [3].

This study has several limitations. Firstly, both RCT and retrospective studies were included in the analysis. Meta-analysis of RCT studies produced high-level evidence, but there were only three RCT studies, with small sample sizes, which compared PTBD and EBD. Because of that, we also included retrospective studies, which were all evaluated as high-quality, and proceeded to conduct stratified analysis according to the study design. Secondly, there is also a significant lack of homogeneity between the different studies with respect to therapeutic success rate and occurrence of most of the complications. Thirdly, publication bias may be a factor, resulting from inclusion of studies only written in English, and inclusion of studies concerning small sample size, which likely provide results that favor PTBD.

## Conclusion

This meta-analysis indicates certain advantages for both PTBD and EBD. In the clinical practice, it is advised to choose specifically either PTBD or EBD, based on location of obstruction, purpose of drainage (as a preoperative procedure or a palliative treatment) and level of experience in biliary drainage at individual treatment centers.

## Abbreviations

CI: Confidence interval
EBD: Endoscopic biliary drainage
MeSH: Medical subject heading
ORs: Odds ratios
PTBD: Percutaneous trans-hepatic biliary drainage
RCTs: Randomized controlled trials
RFA: Radiofrequency ablation
TIAB: Title/abstract
